# Preparation and characterization of novel anti-inflammatory biological agents based on piroxicam-loaded poly-ε-caprolactone nano-particles for sustained NSAID delivery

**DOI:** 10.1080/10717544.2020.1716881

**Published:** 2020-02-03

**Authors:** Azizeh Rahmani Del Bakhshayesh, Abolfazl Akbarzadeh, Alireza Alihemmati, Hamid Tayefi Nasrabadi, Azadeh Montaseri, Soodabeh Davaran, Ali Abedelahi

**Affiliations:** a Drug Applied Research Center, Tabriz University of Medical Sciences, Tabriz, Iran;; b Department of Tissue Engineering, Faculty of Advanced Medical Sciences, Tabriz University of Medical Sciences, Tabriz, Iran;; c Student Research Committee, Tabriz University of Medical Sciences, Tabriz, Iran;; d Department of Nanotechnology, Faculty of Advanced Medical Sciences, Tabriz University of Medical Sciences, Tabriz, Iran

**Keywords:** Piroxicam, intra-articular administration, drug delivery, PCL nanoparticle, collagen

## Abstract

Piroxicam (PX), a main member of non-steroidal anti-inflammatory drugs (NSAIDs), is mainly used orally, which causes side effects of the gastrointestinal tract. It also has systemic effects when administered intramuscularly. Intra-articular (IA) delivery and encapsulation of PX in biodegradable poly-ε-caprolactone (PCL) nanoparticles (NPs) offer potential advantages over conventional oral delivery. The purpose of this study is the development of a new type of anti-inflammatory bio-agents containing collagen and PX-loaded NPs, as an example for an oral formulation replacement, for the prolonged release of PX. In this study, the PX was encapsulated in PCL NPs (size 102.7 ± 19.37 nm, encapsulation efficiency 92.83 ± 0.4410) by oil-in-water (o/w) emulsion solvent evaporation method. Nanoparticles were then characterized for entrapment efficiency, percent yield, particle size analysis, morphological characteristics, and *in vitro* drug release profiles. Eventually, the NPs synthesized with collagen were conjugated so that the NPs were trapped in the collagen sponges using a cross-linker. Finally, biocompatibility tests showed that the anti-inflammatory agents made in this study had no toxic effect on the cells. Based on the results, it appears that PX-loaded PCL NPs along with collagen (PPCLnp-Coll) can be promising for IA administration based on particulate drug delivery for the treatment of arthritis.

## Background

Piroxicam (PX), a chemical called 4-hydroxyl-2-methyl-N-2-pyridinyl-2H-1,2,-benzothiazine-3-carboxamide 1,1-dioxide, and the molecular formula C_15_H_13_N_3_O_4_S is a potent member of non-steroidal anti-inflammatory drug (NSAID) family that exhibits anti-inflammatory, analgesic, and antipyretic activity (Saganuwan, 2016; Chantasart et al., 2018). PX is soluble in most organic solvents, water, and dilute acid (Saganuwan, 2016). PX is employed for the treatment of musculoskeletal pains, especially for articular problems, including osteoarthritis and rheumatoid arthritis. In the use of PX compared to other NSAIDs, only low doses are needed to achieve a therapeutic effect. PX also shows deep localized tissue penetration, which is mainly delivered through the systemic circulation (Saganuwan, 2016). These physicochemical and pharmacokinetic properties make PX an ideal candidate for particulate drug delivery system (Mustapha et al., 2016). However, due to its low molecular weight and also lipophilicity, after topical administration, it shows the highest plasma concentration compared to other NSAIDs (Saganuwan, 2016). Also, PX therapy is usually effective as an oral treatment, but it has many side effects such as gastrointestinal ulceration, renal disorder, and bleeding perforation. However, the most common side effects of PX are peptic ulcers and gastritis (Trivedi et al., 2015). Therefore, in recent years, intra-articular (IA) administration of different therapeutic agents, especially NSAID has been clinically investigated (Kim et al., 2016). Direct IA delivery of active agents to target tissues increases the chance of tissue treatment, even with lower doses, while also having fewer undesirable adverse effects (Bannuru et al., 2015). NSAID is the common IA therapeutic for osteochondral defects in joints (He et al., 2017). Since these molecules can easily be removed from the synovial cavity and transmitted to the bloodstream, unfortunately, their therapeutic activity is often temporary (Kim et al., 2016). This is due to the structure of the outer synovial membrane, in which the synoviocytes form a discontinuous layer containing intercellular gaps (0.1–5.0 μm) (Knight & Levick, 1984). One of the solutions to prolonged local treatment with NSAIDs is the use of IA sustained drug delivery system (Zhang et al., 2018). Most important one of these systems is polymer-based nanoparticle (NP) system that acts as drug carriers to decrease the rapid withdrawal of different therapeutic agents such as drugs from the synovial cavity and increase drug retention time in the joints after IA administration (Mitragotri & Yoo, 2011; Kim et al., 2015; Asadi et al., 2019; Brown et al., 2019). Between the various biodegradable polymers, poly-ε-caprolactone (PCL) has been considered as a drug carrier due to its good properties, such as biodegradability, biocompatibility, and high permeability to micro-molecular drugs (Mei et al., 2018; Zupančič et al., 2018; Asadi et al., 2019). Also, the mild and long-lasting PCL degradability is another good feature that makes it a suitable vehicle for sustained delivery of PX (Manoukian et al., 2018). On the other hand, the use of collagen that is able to absorb, associate and interact with NPs, has a great impact on their biological behavior (Kandamachira et al., 2015). Collagen is one of the most abundant components of the extracellular matrix, which has unique biological properties and results in excellent interaction with cells (Rahmani Del Bakhshayesh et al., 2018; Saghati et al., 2018; Del Bakhshayesh et al., 2019; Kayal et al., 2019; Taghipour et al., 2019). Since the collagen degradation rate is high and its mechanical strength is weak, the appropriate cross-linker should be used for its usage in a variety of applications, including drug delivery in a biological environment (Sallent et al., 2019). It seems that the collagen and NPs conjugation will stabilize them in the synovial cavity and increase biocompatible functionalities of NPs for further environmental interactions. Also, since NPs, smaller than 250 nm rapidly leave the synovial cavity (Kim et al., 2016), translocation of NPs to collagen composites can be a solution to this problem.

According to the above fact, in this study, with the aim of reducing the systemic exposure and, in addition, long-term retention of PX in the synovial cavity, an attempt has been made to formulate an NP delivery system based on PCL and collagen for PX delivery. PX-loaded PCL NPs were prepared by emulsification and solvent evaporation technique and then were characterized in terms of physical and chemical structure, particle size, loading amount of drugs, and *in vitro* release profile. After that, in order to embed NPs in the polymeric bed, and its continuous release, collagen was used and the PX-loaded PCL NP-Collagen composite was prepared and characterized.

## Materials and methods

### Materials

Collagen type I, stannous 2-ethyl hexanoate (stannous octoate, Sn(Oct)_2_) and MTT were purchased from Sigma-Aldrich (Chemical Co., St. Louis, MO). Glutaraldehyde (25% aqueous solution), and all the solvents were purchased from Merck Chemical Co. (Darmstadt‎, Germany). Poly-ε-caprolactone (PCL) was synthesized from ε-caprolactone (ε-CL) monomers purchased from Merck Chemical Co. (Darmstadt‎, Germany). Also, fetal bovine serum (FBS), DMEM, Pen/Strep, and trypsin were purchased from Bio idea. Polymer specifications are presented in Table 1.

**Table 1.** Physicochemical characteristics of the main materials used in this study.

Materials	Formula	Average molecular weight
Poly-ε-caprolactone		*M*_n_ = 10,000 g/mol *M*_w_ = 14,000 g/mol
Collagen		*M*_w_ = 300,000 g/mol
Piroxicam (PX)		*M*_w_ = 331.35 g/mol

### Piroxicam quantification

In order to solubility and release studies, a 100 µg/ml PX stock solution was prepared in distilled water and then diluted 10-fold with either distilled water. By this way, different solutions of PX with concentrations ranging from 5 to 20 µg/ml were then prepared. Piroxicam absorbance was measured at 353 nm in cuvettes (Hellma, Müllheim, Germany) by using UV-visible spectrophotometer (Cecil Aquarius CE 7250; Bio Aquarius, Cecil, UK), with distilled water used as a reference.

### Preparation of piroxicam-loaded PCL nanoparticles

According to before research (Rahmani Del Bakhshayesh et al., 2018), PCL polymer was synthesized by ROP method from ε-CL in the presence of a stannous octoate (Sn(Oct)_2_) as a catalyst. In this regard, in order to initiate the polymerization, Sn(Oct)_2_ was added to ε-CL under a magnetic stirring condition and then exposed to N_2_ gas and 130 °C for six hours. The synthesized polymer was then purified using DCM and diethyl ether. The polymer was first dissolved in DCM, and then the mixture precipitated in a diethyl ether in the cold bath. After the polymer product was dried at the laboratory temperature, its weight was measured and then the degree of polymerization was calculated. Fourier transform infrared (FTIR) spectroscopy was used to analysis the chemical structure of synthesized PCL polymer. In this regard, the polymer was mixed with potassium bromide and pressed to disk. Then, an FTIR spectrometer (Equinox 55 LS 101, Bruker, Bremen, Germany) was used in taking infrared spectra of PCL polymer in a definite range (400–4000 cm^−1^).

Blank and PX-loaded PCL NPs (PCLnp and PPCLnp) were prepared using the emulsion-solvent evaporation (ESE) technique. Typically, PX (20 mg/ml) was dissolved in DCM (15 ml) containing PCL (0.5 g) dissolved in it. This PCL–DCM–PX solution was then mixed with 40 ml water containing PVA (0.5%, w/v) solution. For this purpose, after placing the PCL–DCM–PX solution in a glass syringe and adding dropwise to PVA solution under stirring (800 rpm), the specimens were placed in an ice bath and were sonicated for 3 min (13 W, 40% amplitude). This was done by a microtip probe sonicator (SYCLON, Ningbo, China) for different time intervals (for optimization purpose) at 55 W to yield oil in water emulsion. The solution was then magnetically stirred at 800 rpm for five hours at room temperature in order to evaporate of DCM. NP dispersion was then centrifuged at 10,000×*g* (Hettich, Zentrifugen, Tuttlingen, Germany) for 20 min. Then, in order to obtain the amount of non-encapsulated PX, by an indirect method, supernatants were collected and their absorbance was measured by UV-visible spectrophotometer (described in Section ‘Piroxicam quantification’). In the next step, the particles were washed with deionized distilled water and frozen at –80 °C and then were placed in a freeze-dryer (Telstar-LyoQue). After 24 hours, dried NPs were obtained that were capped until use. In order to establish a control group on the stage of drug release analysis and comparing the release of PX from the treated polymer and the NPs, PX just dissolved in a solution of the PCL polymer at the same ratio and freeze dried.

NP recovery was calculated using the equation below:

$$\text{NP recovery}=\frac{\text{the mass of the NPs after freeze}-\mathrm{drying}}{\text{the mass of polymer or polymer with piroxicam}}\times 100\%$$

Also, the amount of the drug encapsulated and EE in the NPs was taken using the equation below:

$${\text{Drug}}_{(\text{encapsulated})}={\text{Drug}}_{(\text{total})}–{\text{Drug}}_{(\text{non}-\text{encapsulated})}$$

$$\mathrm{EE}=\frac{\mathrm{drug}\;(\mathrm{encapsulated})\;}{\text{the total amount }(\mathrm{mass})\text{ of piroxicam}}\times 100\%$$

### Size, zeta potential, and polydispersity of nanoparticles

Dynamic light scattering (DLS) (Malvern Instruments, Malvern, UK) was used to measure the particle size distribution, zeta potential, and polydispersity index of the PCL NPs and PPCL NPs. In order to achieve these data, the dried powder of NPs was suspended in water and sonicated shortly before measurement to prevent clumping. Size distribution and mean diameter of the gained homogeneous suspension were then determined. The NPs were then analyzed for their surface potential charge employing a Zetasizer (Malvern Instruments, Malvern, UK).

### Preparation of biocompatible piroxicam-loaded PCL nanoparticle agents

To improve biological behaviors of NPs, PCL NP-Collagen (PCLnp-Coll) and PX-loaded PCL NP-Collagen (PPCLnp-Coll) composites were prepared. For this purpose, NaOH was used for surface hydrolyzation of NPs. Then, the 10 mg/ml of type I collagen at the pH of 4 and the NPs stock solution was prepared. Hydrolyzed NPs were then immersed in a collagen solution for 1 h at room temperature and glutaraldehyde (1%) was used as a cross-linker. Then, they were washed in distilled water to eliminate the free collagen and freeze-dried for use. Since uncross-linked glutaraldehyde is toxic, free glutaraldehyde should be eliminated. Therefore, samples were washed several times with distilled water and the glycine 0.1 M was used to quench glutaraldehyde residues.

### Fourier transform infrared spectroscopy

Fourier transform infrared spectroscopy was employed to study the chemical structure of PCLnp, PPCLnp, PCLnp-Coll composite, and PPCLnp-Coll composite. For this purpose, compressed discs containing potassium bromide and NPs were prepared, and then the FTIR spectrum of the samples was analyzed in the range of 400–4000 cm^−1^ by the FTIR spectrometer (Equinox 55 LS 101, Bruker, Bremen, Germany).

### Characterization by differential scanning calorimetry

Analysis of the physical status (the thermal characterization) of PCLnp, PPCLnp, PCLnp-Coll, and PPCLnp-Coll groups, was measured by differential scanning calorimeter thermogram analysis (DSC, Netzsch, Selb, Germany). In this method, samples (10 mg) were placed inside a standard aluminum pans with lids and then sealed. Each of the samples was then heated from 30 °C to 380 °C with a heat increase rate of 5 °C·min^−1^. By this method, any possible variation in the melting temperature of the groups was evaluated.

### *In vitro* release of piroxicam from nanoparticles

The release studies of PPCLnp and PPCLnp-Coll groups were done in triplicate. In order to evaluate the release of PX from these groups, the dialysis bags (exclusion size 1.4 × 10^4^ MW) containing 20 mg PX-loaded PCL NPs alone or with collagen was placed in a falcon tube containing 20 ml PBS (0.2 M, pH = 7.4) and incubated at 37 °C in a shaker with horizontal shaking (50 rpm). Pure PX and treated PCL polymer was used as a control group. At specified time intervals, the released medium were changed with 20 ml fresh PBS. PX concentration was then measured through the absorbance of elution medium at 353 nm using a UV-Vis spectrophotometer. The following equation was used to analyze the data related to the release:

$$\text{Cumulative amount released }(\%)=\frac{\text{the amount of piroxicam released at time }t}{\text{the amount of piroxicam released at infinity }}\times 100$$

$$W_{t}{}_{(\text{the}\;\text{amount}\;\text{of}\;\text{piroxicam}\;\text{released}\;\text{at}\;\text{time}\;t)}=\;W_{\infty \;(\text{the}\;\text{amount}\;\text{of}\;\text{Piroxicam}\;\text{released}\;\text{at}\;\text{infinity})}\left(1-e^{\mathrm{-kt}}\right)$$

where *k* is the release rate constant (Corrigan et al., 2006). In this study, at the beginning of the experimental studies (*t* = 0 min), *W* is assumed to be zero (*W* = 0 µg/mg), which means that no drug was released at zero time.

### Swelling behavior and dissolution assessment

The swelling properties of the PCLnp, PPCLnp, PCLnp-Coll, and PPCLnp-Coll groups were evaluated by measuring the equilibrium water uptake of samples with predetermined weight (*W****′***) and incubated in PBS (pH = 7.4) at 37 °C. At specified times (3 h, 6 h, 12 h, and 24 h), all samples were taken out from the buffer and excess water was then carefully removed. The swollen NPs were then weighed (*W****″***) and dried at 60 °C in the oven until the weight of the dry specimens was unchanged. The weight of dried NPs was recorded (*W****‴***). Each experiment was repeated three times and the average value was used to calculate the swelling capacity (%) and dissolution rate by the following equations.

$$\text{Swelling capacity }(\%)=\frac{W\prime \prime -W^{\prime \prime \prime }}{W^{\prime \prime \prime }}\times 100$$

$$\text{Dissolution rate}\;(\%)=\frac{W\prime -W^{\prime \prime \prime }}{W\prime }\times 100$$

### Surface morphology of nanoparticles and nano-composites

The surface morphology and particle size of PCLnp, PPCLnp, and their Coll hybrid structures were analyzed by a scanning electron microscope (MIRA3, Tescan, Brno, Czech Republic). For this purpose, the specimens were first dried by freeze drying, so that any moisture content was removed. Then, dried specimens are spread on an aluminum plate and coated with a thin layer of gold under vacuum and visualized by SEM.

### Cytocompatibility studies

PCLnp, PPCLnp, and their Coll hybrid structures were placed in the 96-well plate and sterilized by UV radiation, and to remove any residual solvent, they were washed thrice with PBS. AT-MSCs were cultured in DMEM supplemented with 10% FBS and 1% antibiotics at 37 °C and 5% CO_2_. After the confluency reached 80%, the cells were trypsinized and seeded onto PCLnp, PPCLnp, PCLnp-Coll, and PPCLnp-Coll at a cell density of 10^4^ cells per well and incubated at 5% CO_2_ and 37 °C. Finally, the viability of the cells was assessed using the MTT assay after the 24, 48, and 72 hours of culture.

### Statistical analysis

All experiments were done in triplicate, and data are represented as mean ± SD (*p<.05, **p<.01, ***p<.001, and ****p<.0001). The statistical significance of the differences among samples was employed using GraphPad Prism 6.0 (GraphPad Software, La Jolla, CA). One-way analysis of variance (ANOVA) was used for NP recovery, EE, kinetics of PX release, swelling and dissolution data, and *t* test was also used for particle size, polydispersity index, and zeta potential data. Two-way ANOVA analysis was also used for cytocompatibility study.

## Results and discussion

### Characterization of synthesized polymer

**Figure 1.**
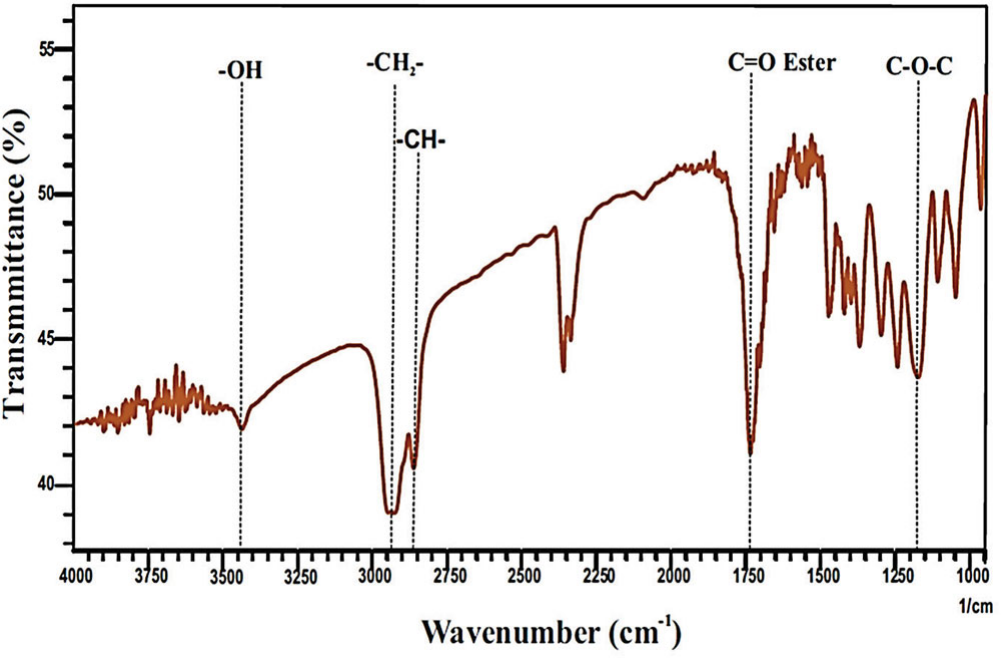
FTIR analysis of PCL polymer.

In this work, PCL polymer was synthesized through ring-opening polymerization (ROP) of caprolactone and stannous octoate (Sn(Oct)_2_) was used as a catalyst. Degree of polymerization of polymer by this method was about 94%. To investigate the chemical structure of synthesized PCL polymer, FTIR spectroscopy was used. According to the FTIR spectrum (Figure 1), the peaks shown in 3450, 2950, 2850, 1750, and 1150–1200 are related to O–H, CH_2_, C–H, C=O, and C–O–C, respectively. The information obtained from this section confirms the accuracy of the PCL polymer synthesis.

### Emulsification solvent evaporation method

**Figure 2.**
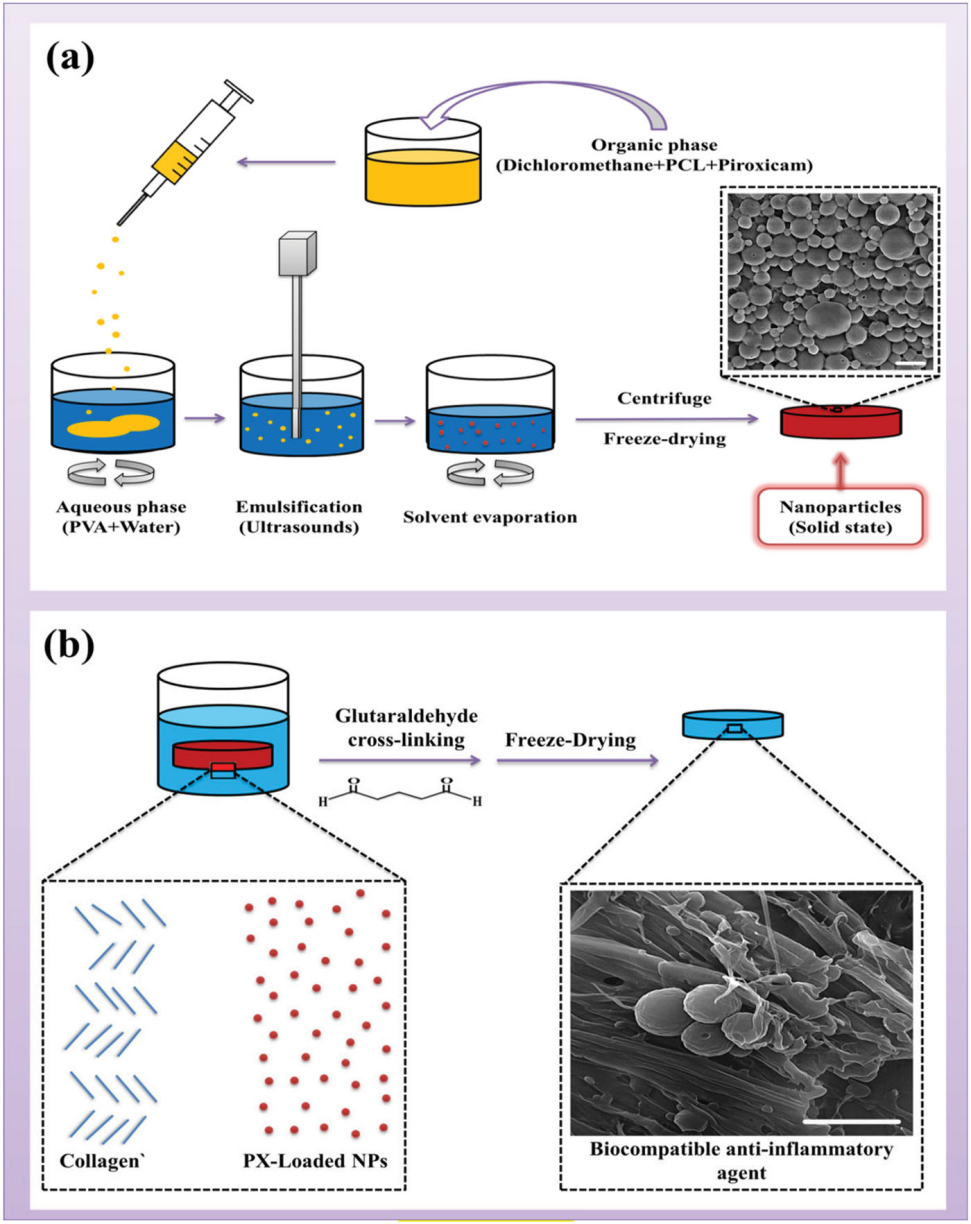
(a) Schematic illustration of PCL nanoparticle fabrication process. (b) Preparation of biocompatible piroxicam-loaded PCL nanoparticle agents.

There are various methods for creation of NPs. Many researchers have used emulsification solvent evaporation (ESE) method to produce nano- and micro-particles (Mamat et al., 2019). For example, in a study conducted by Deng et al., to improve the skin penetration of ibuprofen, ibuprofen nanoparticles (IBU-NPs) with a mean particle size of 216.9 ± 10.7 nm were prepared through the ESE method (Deng et al., 2018). Similarly, we also used this method for the preparation of PCL NPs in the present study. In this method, polyvinyl alcohol (PVA) was used as a surfactant and dichloromethane (DCM) as a solvent. Also, ultrasonication was used and all process factors, such as stirring speed, sonication time and intensity, and quantities of the excipients were set and kept constant. Figure 2(a) shows a schematic diagram of this process. In this way, to ensure proper NPs reconstitution and to their more stability during the freeze drying process, suitable excipients are needed. It has been suggested that PVA may play the role of cryoprotectant and stabilizing agent throughout the lyophilization of NPs. Therefore, PVA acts as an emulsion stabilizer and stabilizes the polymeric NPs in both of the solution state during the emulsification step and in solid state after the freeze drying process. Also, since the PVA bonding affects the hydrophilicity/hydrophobicity of the surface of the polymer particles and hence its digestion (Sahoo et al., 2002), it can accelerate the wetting and destruction of PCL NPs.

### Formation and characteristics of blank PCL NPs, PX-loaded PCL NPs, and composites of blank PCL NPs-Collagen and PX-loaded PCL NPs-Collagen

After synthesis of NPs by the ESE method, the first feature to be investigated is the macroscopic homogeneity through visual observation. In this regard, it was macroscopically observed that all samples were milky-white and homogeneous. Also, the Tyndall effect in nano-suspensions was hardly observed, in which the longer wavelengths are transmitted more than short wavelengths, and the latter is more strongly scattered (Jerlov & Kullenberg, 1953). In addition, dispersion of PX-loaded PCL NPs was bright yellow, due to the presence of PX.

In nanosuspensions known as colloid systems, NPs are thermodynamically unstable and may aggregate after long periods of storage (Süß et al., 2019). On the other hand, a major challenge in the expansion of NPs is the long-term stability. So, the possibility of achieving a solid form is a second important parameter that should be considered when formulating NPs. One of the best approaches to creating solid nano-suspensions with more stability is freeze-drying, which also provides easy handling of them (Rouquette et al., 2019; Sheikhi et al., 2019). After freeze-drying for 24 hours, the texture of the lyophilized particles was similar to cotton. In terms of appearance, the blank PCL NPs after freeze-drying were white and PX-loaded PCL NPs were bright yellow.

**Figure 3.**
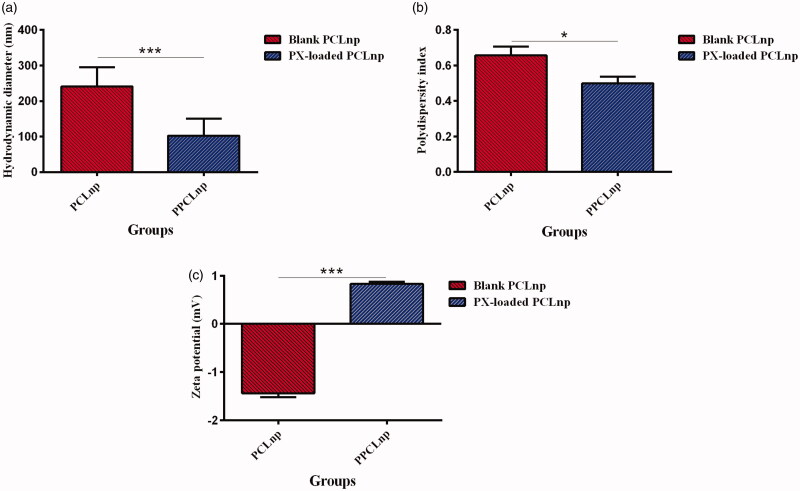
(a) Hydrodynamic diameters of blank and PX-loaded PCL NPs based on DLS measurements and SEM image analysis with ImageJ software. (b) Polydispersity index of blank and PX-loaded PCL NPs. (c) Zeta potential of blank and PX-loaded PCL NPs. Results are expressed as mean values (*n* = 3) ± standard deviation and compared using *t* test.

Since the size of the NPs affects their biopharmaceutical properties and drug release (Kumar et al., 2013), it was investigated using DLS measurements and SEM observations. It was determined by measuring the particle size that the polymeric NPs were successfully synthesized through the ESE method (Figure 3(a)). The particle size gained for PCL NPs was 241.1 ± 20.38 nm and the presence of small amounts of aggregates was also observed. In this regard, it was observed that PX loading on the PCL NPs affected the size of NPs (*p*= 0005). Interestingly, incorporation of PX enhanced the re-dispersibility of PX-loaded PCL NPs compared with blank PCL NPs. The re-dispersed PX-loaded PCL particles were macroscopically homogenous and the particle size of them was 102.7 ± 19.37 nm. The second parameter obtained from the DLS measurements was the polydispersity index. The polydispersity index is the ratio of size deviation to mean particle diameter, and the higher values of which show large variations in particle size (Chopra et al., 2016). PX loading significantly decreased the polydispersity index in the PX-loaded PCL NPs (*p*= 0125) (Figure 3(b)). So, a lower particle diameter and more homogeneous size distribution in PX-loaded PCL particles indicate the improvement of the re-dispersibility of PCL NPs by PX loading. Another important parameter obtained from DLS measurements is the zeta potential, which represents the material charges (Akkus et al., 2019; Gomillion, 2019). As reported several times, PCL, which is an uncharged polymer (Abriata et al., 2019), displayed a negative zeta potential values close to neutral (Figure 3(c)). Zeta potential of PCL NPs was –1.440 ± 0.0600 mV. PX-loaded PCL particles exhibited a slightly positive zeta potential of 0.8330 ± 0.03300 mV. Hence, PX loading significantly affected the zeta potential of NPs (*p*= 0009). However, according to the results, it can be said that both blank and PX-loaded PCL NPs are nearly neutral.

**Figure 4.**
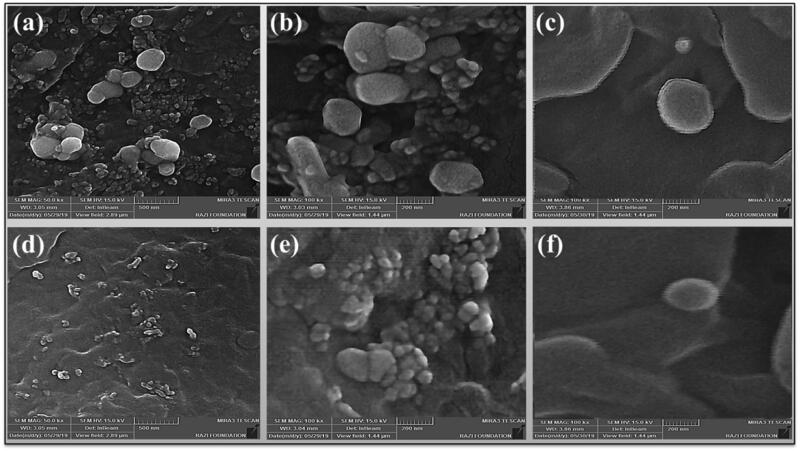
SEM of blank PCL NPs (PCL NPs) (a–c) and PX-loaded PCL NPs (PPCL NPs) (d–f).

Figure 4 shows the SEM images taken from PCL NPs and PPCL NPs. Both blank and PX-loaded PCL NPs were agglomerated (Figure 4(a,b,d,e)). Both PCL NPs and PPCL NPs had spherical shapes, but in some cases, the deformation is also due to the presence of neighboring particles, which was also observed in the images. Also, it should be noted that local heating from the SEM beam can alter particle morphology and destroy them. As seen in the picture, re-dispersibility of PCL NPs has improved with PX loading.

**Figure 5.**
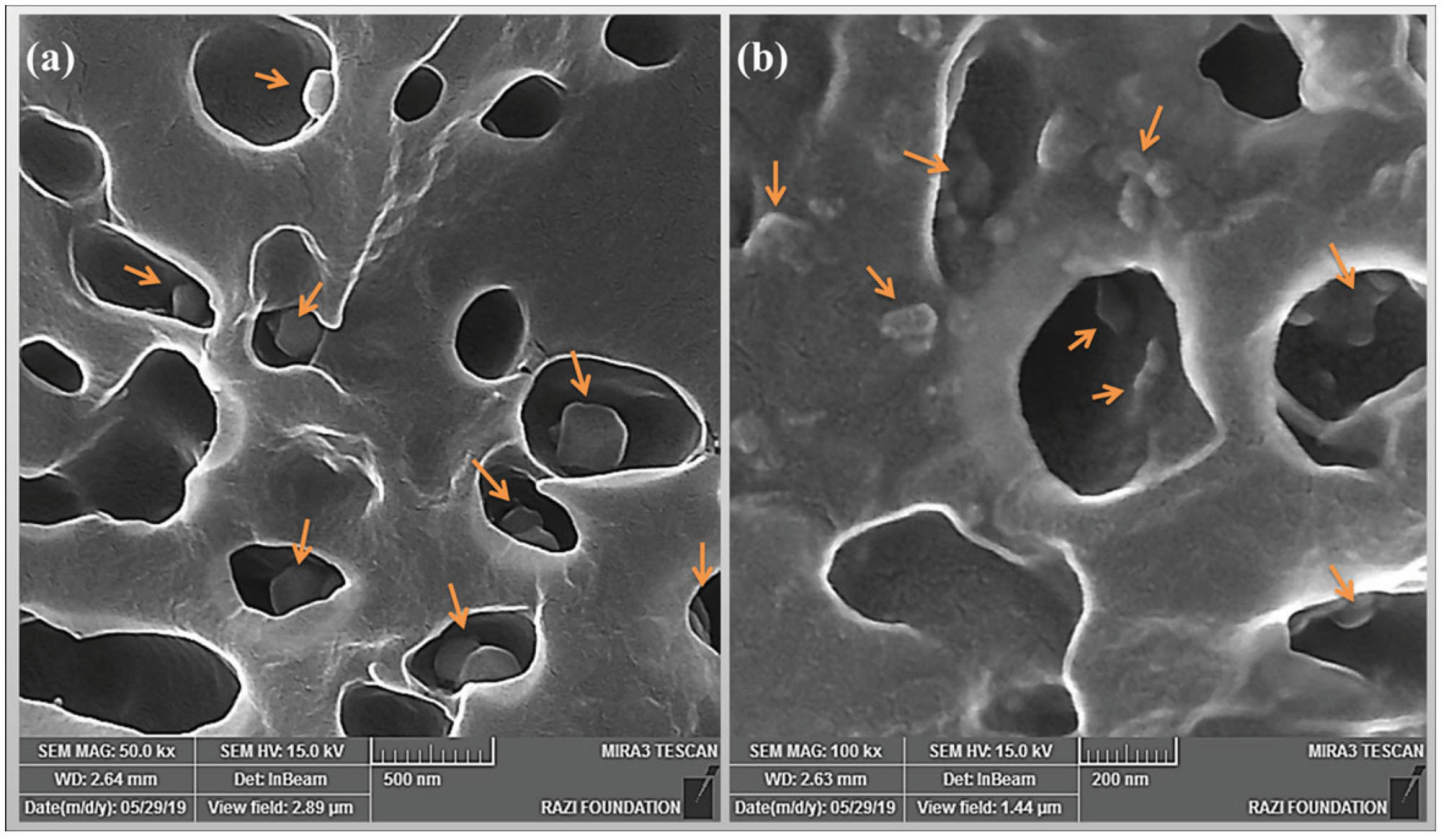
SEM images of the PX-loaded PCL NP-Collagen composite. Scale bar: (a) 500 nm and (b) 200 nm.

In this work, nanoscale particles were created to sustained release of PX, but since these particles can escape from the 0.1 to 5.0 μm pores in the synovial membrane and enter the bloodstream (Kim et al., 2016), in order to prevent that and also to create biocompatible agents, collagen was used. Therefore, biocompatible composites of PCLnp-Coll and PPCLnp-Coll were obtained by immersing PCLnp and PPCLnp in a collagen solution and making covalent connections through a cross-linker (Figure 2(b)). The uniform presence of NPs in a collagen composite can be seen in Figure 5. By creating anti-inflammatory bio-agents consisting of collagen and PX-loaded PCL NPs, the possibility of NPs removal in the early hours from the synovial cavity, through the pores of the synovial outer membrane, will be prevented. Hence, duration of treatment until complete degradation of the composite and then slow release of the NPs and following this, the release of PX will be longer.

**Figure 6.**
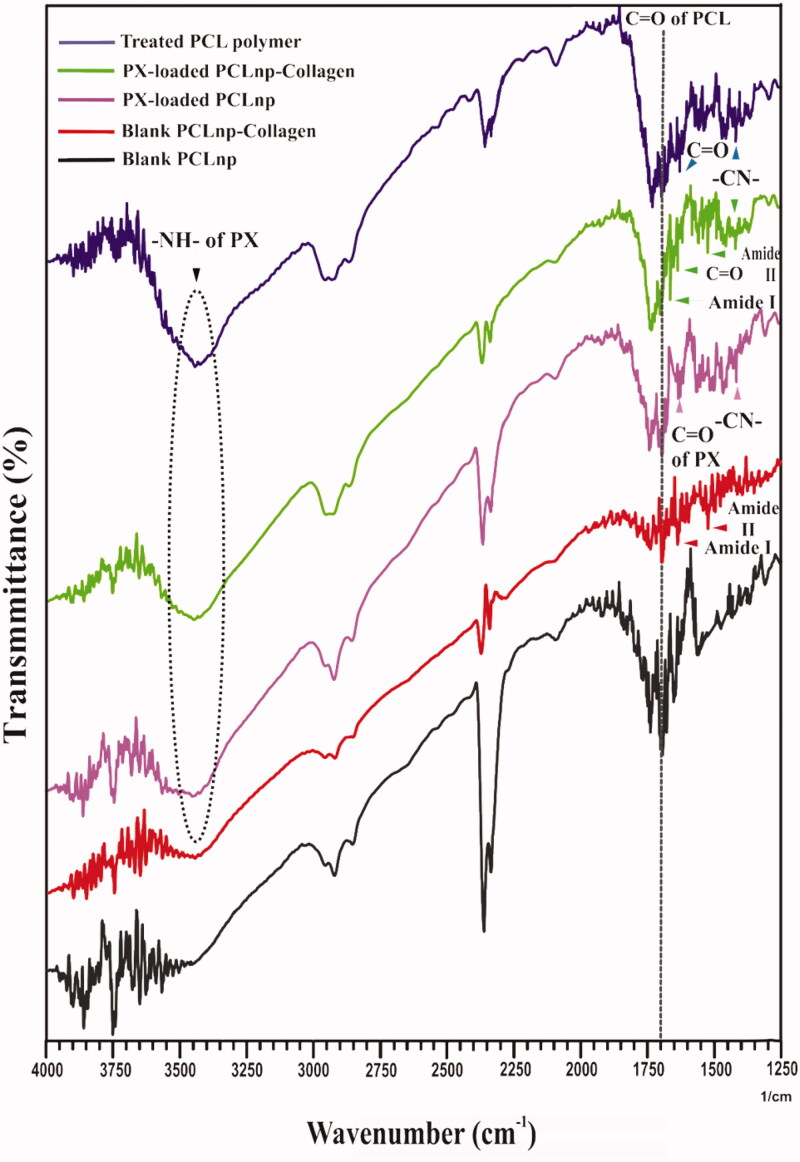
FTIR spectrum of blank PCLnp (PCLnp), blank PCLnp-Collagen (PCLnp-Coll), PX-loaded PCLnp (PPCLnp), PX-loaded PCLnp-Collagen (PPCLnp-Coll) and treated PCL polymer.

Figure 6 shows the FTIR spectroscopy of PCLnp, PPCLnp, treated polymer, PCLnp-Coll, and PPCLnp-Coll, which was performed to investigate the interaction between polymeric NPs and PX. The characteristic FTIR bands of PX at 1400–1450 cm^−1^, 1600–1650 cm^−1^, and 3300–3450 cm^−1^ that are related to C–N, C=O, and N–H, were observed in the FTIR spectra of treated polymer, PPCLnp, PPCLnp-Coll. Also, the strong absorption in 1700 cm^−1^ correspond to C=O of PCL, which was observed in all groups. In the spectrum of the PCLnp-Coll and PPCLnp-Coll composites, the bands around the region at 3300–3450 cm^−1^ are related to the N–H and hydrogen bond of –OH stretching in collagen. This band overlaps in the PPCLnp-Coll’s spectrum with the N–H band of PX. Also, the amide bands (N–H bending at 1525 cm^−1^ for amide II, C=O stretching at 1650 cm^−1^ for amide I) characterized by Coll, was observed in the spectrums of PCLnp-Coll and PPCLnp-Coll.

### Differential scanning calorimetry (DSC)

**Figure 7.**
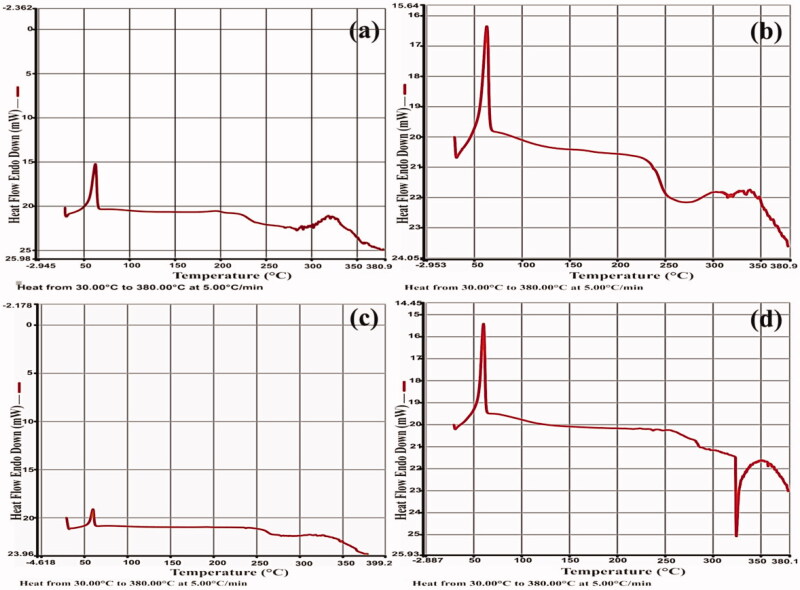
The DSC measurement of the PCLnp (a), PCLnp-Coll (b), PPCLnp (c), and PPCLnp-Coll (d).

Figure 7 displays DSC thermograms of PCLnp, PPCLnp, and composites of PCLnp-Coll and PPCLnp-Coll based on sample weight. DSC estimated the thermal stability profiles of all four main samples. These data are based on the cooling step, starting from the melting state.

PCLnp has an endothermic peak at around 56 °C, which relates to its melting point (Tm). All four groups had a single endotherm around Tm of PCL (Figure 7(a–d)). It has been shown that endothermic denaturation of collagen occurs at about 325 °C (Moraes et al., 2016), which is largely observed for freeze-dried PCLnp-Coll and PPCLnp-Coll.

According to previous studies, we also know that the PX melting temperature is around 199–201 °C (Chantasart et al., 2018). However, the DSC thermograms of PPCLnp (Figure 7(c)) and PPCLnp-Coll (Figure 7(d)) did not show the melting peak in the above mentioned temperature. This indicates the complete dissolution of PX in these samples below the melting temperature of PX. Hence, these samples formed monotectics during the DSC heating process. The DSC curves indicate good thermal properties, despite a change in its thermal stability after adding collagen.

### Nanoparticle recovery and encapsulation efficiency

NP recovery was defined by dividing the mass of PPCL NPs into the mass of PCL polymer and PX. It should be noted that during the different stages of NP formulation, such as the purification step, material transfer is required that can cause NP loss (Umerska et al., 2018). Hence, it is necessary to calculate NP recovery, which is also called the NP yield. In this regard and in order to calculate the NP recovery, for the blank NPs, only the weight of PCL used to generate NPs was desired, but for PX-loaded particles, the initial weight of PX and PCL were required. As seen in Table 2, PX loading in the NPs has had an effect on the NP yield (*p*= 0040). Approximately, 74–77% of PCL NPs were recovered, while the recovery rate of PX-loaded NPs is about 65–69%. The smaller yield obtained for PPCLnp may be due to the presence of an important part of the small size NPs that did not sedimentation during the centrifuge process. This was confirmed in the DLS measurements that the PPCL are the smallest NPs.

**Table 2.** Nanoparticle recovery and encapsulation efficiency.

Formulation	Blank PCL NPs (PCLnp)	PX-loaded PCL NPs (PPCLnp)
NP recovery (%)	75.67 ± 0.8819, *n* = 3	67.00 ± 1.155**, *n* = 3
Encapsulation efficiency (%)	N/A	92.83 ± 0.4410, *n* = 3

Results are expressed as mean values (*n* = 3) ± standard deviation and compared using *t* test.

N/A: not available.

** *p* < .01 versus blank PCL NPs.

Drug loading is a main parameter in the development of an NP-based delivery system because with low loading a significant amount of formulation is required to achieve a therapeutic effect (Tan et al., 2018; Thauvin et al., 2018). Therefore, low drug loading is often a limiting factor in using these systems. Thus, high drug encapsulation efficiency (EE), as a key factor, is essential for making successful formulations. The amount of EE for PCL NPs was 92.83 ± 0.4410%, which means 92.83 ± 0.4410% of PX incorporated within the NPs.

Since this type of drug delivery system is intended for direct sustained release of PX into the IA cavity, it is anticipated that the proper concentration of PX will be provided to produce anti-inflammatory effects through this way (Park et al., 2014).

### Swelling degree and dissolution rate

**Figure 8.**
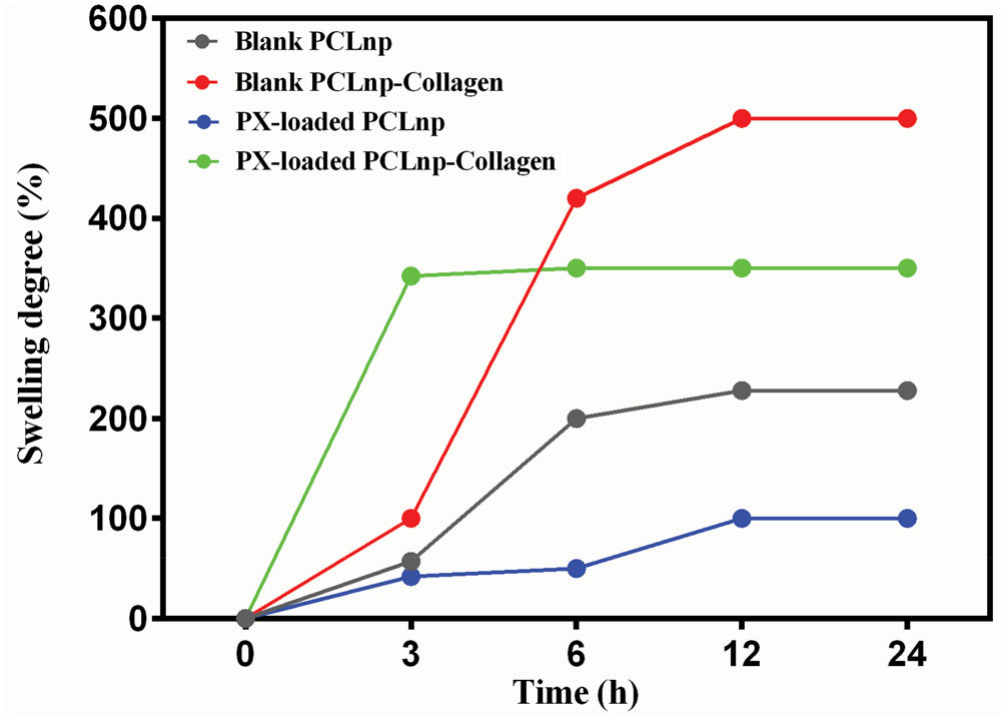
Swelling behavior of PCLnp, PPCLnp, PCLnp-Coll and PPCLnp-Coll with respect to time. Results are expressed as mean values (*n* = 3) ± standard deviation and compared using one-way ANOVA. All samples were immerged in PBS (pH = 7.4) at 37 °C.

Swelling behavior is a key aspect for the NPs that behave as carriers of drugs or different bioactive agents for sustained release (Gopi et al., 2016). There are several ways in which drugs that are in NPs are released (Prasad et al., 2016). One of these methods is the release of drugs due to the erosion of the NPs, which is involved in the delayed release of drugs from NPs made of polymers with prolonged biodegradability such as PCL (Kamaly et al., 2016; Grossen et al., 2017). Another way is the diffusion of drugs that is directly related to the wettability and swelling of the NPs, as the release of the drug occurs following the penetration of water into NPs (Prasad et al., 2016). As can be seen in Table 3 and Figure 8, since NPs were made of PCL polymer in this study, they had a little swelling degree due to their hydrophobic nature. Also, since PPCLnp was loaded with PX, they showed less swelling than blank NPs. Composites of PCLnp-Coll and PPCLnp-Coll showed the degree of swelling more than PCLnp and PPCLnp, due to the presence of collagen, which is highly hydrophilic. As shown in Figure 8, the composite of PPCLnp-Coll has reached the maximum swelling rate after three hours of immersion in PBS and then remained constant. In contrast, the composite of PCLnp-Coll had the highest degree of swelling due to the presence of collagen and blank NPs in the structure. Finally, according to Table 3 and Figure 8, composites of PCLnp-Coll and PPCLnp-Coll have the best swelling degree after 24 hours of immersion in the PBS (*p*< 0001).

**Table 3.** The swelling degree and mass loss percent of PCLnp and PPCLnp during 24 h before and after blending with collagen through glutaraldehyde cross-linking in PBS (pH = 7.4) at 37 °C.

Sample	PCLnp	PCLnp-Coll	PPCLnp	PPCLnp-Coll
Swelling degree (%)	232.7 ± 8.969	488.3 ± 6.009	103.0 ± 9.074	348.3 ± 4.410
Mass loss percent (%) (dissolution rate)	27.59 ± 0.4313	3.820 ± 0.4779	16.07 ± 0.2395	2.833 ± 0.7265

**Figure 9.**
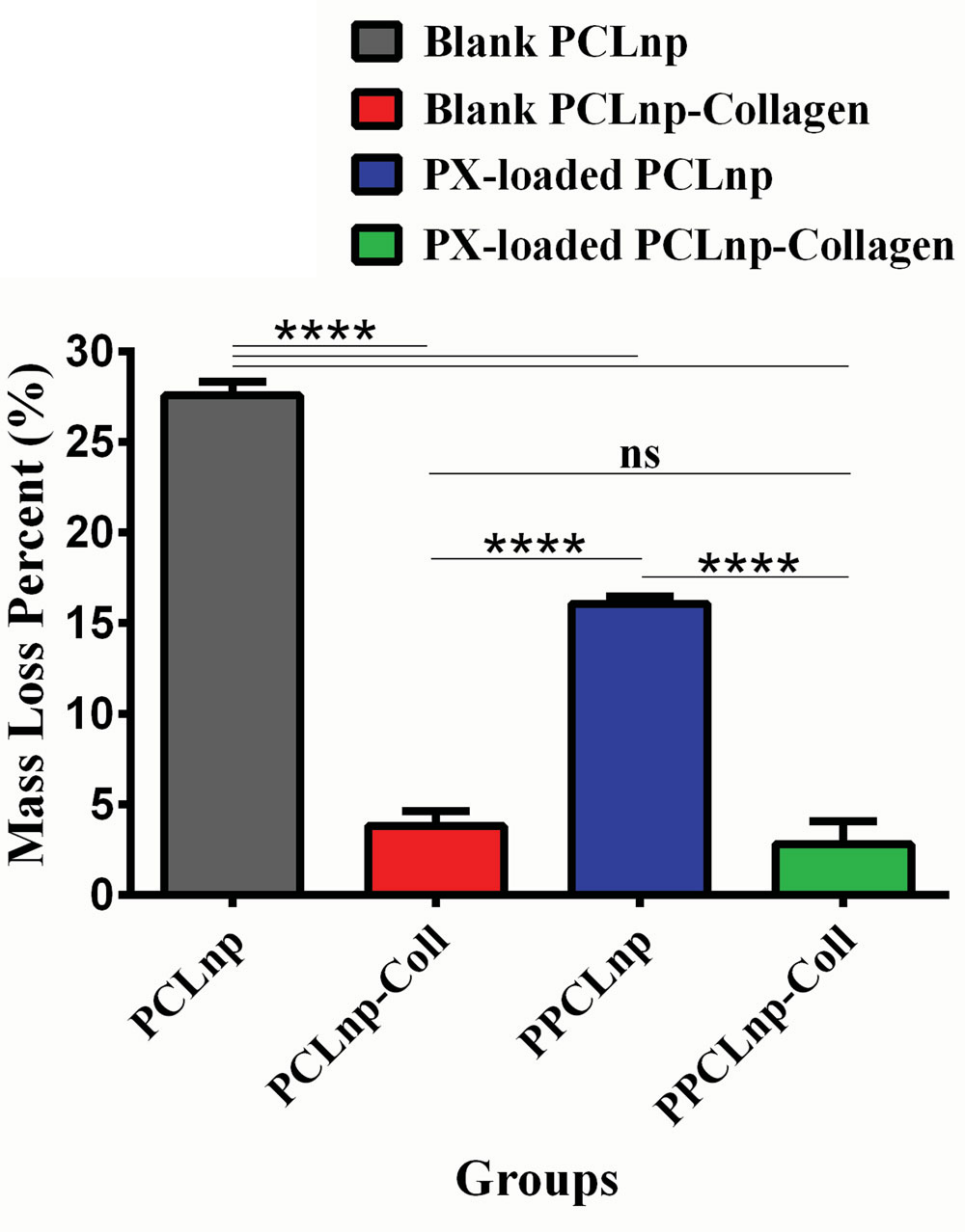
Weight loss of PCLnp, PPCLnp, PCLnp-Coll, and PPCLnp-Coll after 24 hours incubation. Results are expressed as mean values (*n* = 3)±standard deviation and compared using one-way ANOVA. Horizontal line and ‘****’ show significant difference between different groups (*p*<.0001) and ‘ns’ indicates no significant difference among groups. All samples were immerged in PBS (pH = 7.4) at 37 °C.

Dissolution of NPs is critical during drug delivery, since along with the diffusion of drugs, erosion of NPs is also helpful in drug release (Kamaly et al., 2016). Figure 9 shows weight loss of PCLnp, PPCLnp, PCLnp-Coll, and PPCLnp-Coll after 24 hours incubation in the PBS (37 °C). It is completely evident that after 24 hours of incubation, PCLnp had a significantly higher hydrolytic degradation with a dissolution rate of 27.59 ± 0.4313%. According to Table 3, dissolution rate of PCLnp-Coll, PPCLnp, and PPCLnp-Coll was 3.820 ± 0.4779%, 16.07 ± 0.2395%, and 2.833 ± 0.7265%, respectively. These data indicate that both drug loading and collagen treatment had a significant effect on the dissolution rate, as all three cases of PCLnp-Coll, PPCLnp, and PPCLnp-Coll had a significantly lower weight loss than PCLnp (*p*< 0001). It was also found that due to the collagen combination with NPs and the use of cross-linker, a significant decrease in dissolution rate of PCLnp-Coll and PPCLnp-Coll was observed (*p*< 0001). The reason for this is the use of glutaraldehyde cross-linker which has created a compact and stable composite.

### *In vitro* release studies

*In vitro* experiments were done to determine the effect of composite on the kinetics of PX release from NP polymers. PX is more soluble at pH 7.4 than other pH buffers due to its weak acidic properties. According to previous studies, PX dissolves in the pH 7.4 buffer, about 10 times more than the pH 6 buffer (Abdulkarim et al., 2010). Therefore, given that higher solubility in buffer with pH 7.4 can accelerate membrane drug delivery, drug release studies were conducted in the present study in PBS buffer with pH 7.4 and body temperature (37 °C).

**Figure 10.**
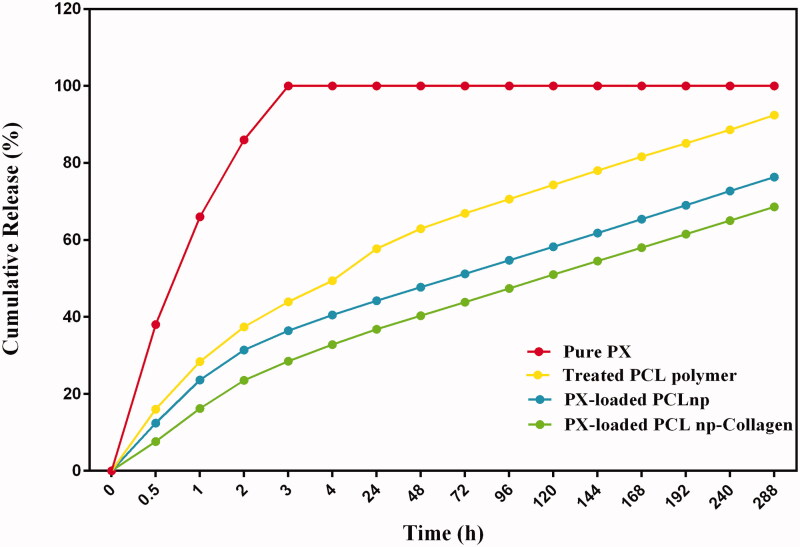
Cumulative release profiles of PX from PX-loaded PCL NP, composite of PX-loaded PCL NPs-Collagen, treated PCL polymer and suspension of PX raw material into PBS for a period of 12 days. Two control groups, including equivalent free drug (PX) dispersed in PBS buffer, and treated PCL polymer (the suspension of PX raw material), were used. Samples were immerged in PBS (pH = 7.4) at 37 °C, and under a shaking 50 rpm.

**Figure 11.**
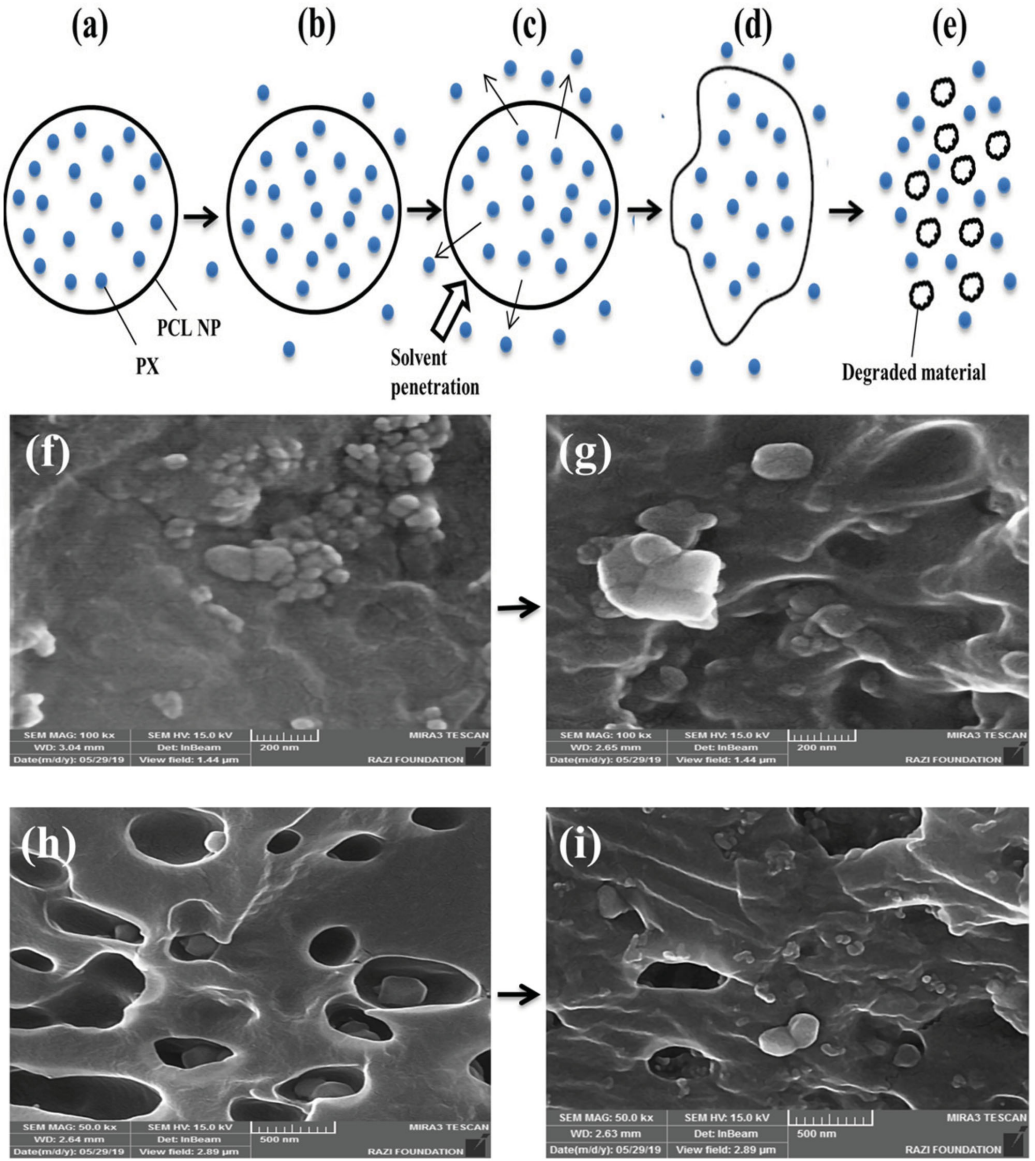
Schematic illustration of diffusion/degradation-controlled PX release mechanism through PCL NPs (a–e) and correlation with SEM images of PPCLnp (f–i): (a) PPCLnp containing PCL NP and PX. (b) Releasing of PX immediately after incubation from the surface. (c) Releasing of PX through diffusion. (d) Releasing of PX through the degradation of NP. (e) Releasing of trapped PX rapidly after disintegration of NP. (f) SEM image of PPCLnp immediately after incubation. (g) SEM image of PPCLnp 12 days after incubation. (h) SEM image of PPCLnp-Coll bio-agent immediately after incubation. (i) SEM image of PPCLnp-Coll bio-agent 12 days after incubation.

Among the studied groups, with the exception of equivalent free drug (PX) dispersed in PBS buffer, which was the control group and showed a release rate of 100% in the early hours, the highest rate of release was observed in the treated polymer (the suspension of PX raw material) (Figure 10). After 12 days (288 h) of incubation, 89.23 ± 2.202% of the drug were released from the treated PCL polymer. It was also observed that PX-loaded PCLnp and PX-loaded PCLnp-Coll released 76.87 ± 1.727% and 63.97 ± 2.505% of their drug after 12 days, respectively (Table 4). A burst release of the PX in the early hours was not seen in the samples of PX-loaded PCLnp and PX-loaded PCLnp-Collagen. This reflects the good texture of PX-polymer and also shows the effective loading of the PX in the NPs, as poor absorption of PX at the NP surface results in early drug release (Rizvi & Saleh, 2018), which was not evident in the present study. However, it is certainly not possible to say that no drug is attached at the surface of NPs. So, along with the main methods of drug releasing, including drug diffusion from the matrix and matrix surface erosion, the release of drug in the early hours can be as a result of the release of PXs attached to the surface of the NPs, but not predominantly. Of course, due to the SEM images taken from the incubated specimens for 12 days for drug release studies, no significant deformation indicating the degradation and erosion of NPs was observed, probably due to the polymer type used to make the NPs (PCL) (Figure 11). Regarding this, it is likely that the release of the PX from the NPs in the present study was due to drug diffusion from the matrix rather than matrix erosion. In addition, the faster release of the drug from the NPs to the environment requires water penetration into the NPs (Carson et al., 2016), but since the nature of the PCL is hydrophobic, penetration of water into the PCL has occurred over a longer period of time, leading to the slow and sustained release of the drug. In addition, as shown in Figure 11, the composite of PPCLnp-Coll was exposed to surface erosion after 12 days of incubation and subsequently the PPCL NPs embedded in it were exposed to the medium. As can be seen from Figure 10, it can be said that the PX release from the PPCLnp-Coll is longer than the PPCLnp alone.

**Table 4.** Drug release of pure PX, treated PCL polymer, PX-loaded PCLnp and PX-loaded PCLnp-Collagen after 12 days incubation in PBS (at pH 7.4, and temperature 37 °C), and under a shaking 50 rpm.

Sample	Pure PX	Treated PCL polymer	PX-loaded PCLnp	PX-loaded PCLnp-Collagen
Drug release %	100.0	89.23 ± 2.202, *n* = 3	76.87 ± 1.727, *n* = 3	63.97 ± 2.505, *n* = 3

According to the results of this study, prolonged release over a period of 12 days was observed in the PX-loaded PCLnp and PX-loaded PCLnp-Collagen. In particular, the composite of PX-loaded PCLnp-Collagen with covalent connections through aldehyde cross-linker had the lowest dissolution rate, which was why it has a more prolonged release (*p*< 0001). Accordingly, the structures constructed in this study appear to be able to suppress local inflammation in the first weeks of implantation. Thus, in addition to preventing systemic side effects of PX, these agents may also increase the chance of repairing damaged cartilage in cartilage diseases. Therefore, they can be used to improve the healing performance of engineered scaffolds due to their anti-inflammatory properties in cartilage tissue engineering. However, the concentration of drug loaded in the NPs in the later phases of the study will vary according to the standards available *in vivo* drug delivery systems and the species under investigation, which requires animal studies.

### Cytotoxic assay

**Figure 12.**
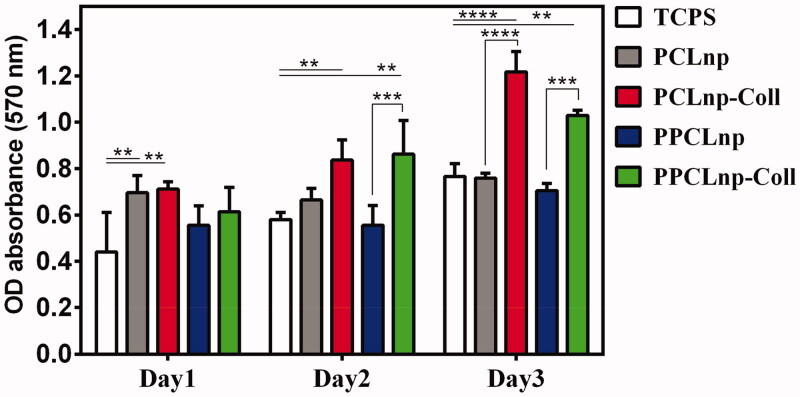
Cytocompatibility of PCLnp, PCLnp-Coll, PPCLnp, and PPCLnp-Coll studied by the MTT assay. Results are expressed as mean values (*n* = 3)±standard deviation and compared using two-way ANOVA.

For the potential use of bio-agents as part of the structure of implantable or injectable scaffolds in cartilage regeneration, evaluation of cell viability is essential to evaluate the toxicity of bio-agents. In this study, adipose tissue-derived mesenchymal stem cells (AT-MSCs), which have been widely used in osteoarthritis studies due to their immune modulatory and immunosuppressive properties (Yanez et al., 2006; Harrell et al., 2019), were cultured on PCLnp, PPCLnp, PCLnp-Coll, and PPCLnp-Coll structures for 24, 48, and 72 hours. Also, MTT assay was used to evaluate cell viability in these bio-agents (Figure 12). Cells cultured in TCP as control were gradually proliferated over 3 days of incubation. For all groups, there was a difference in cell viability after 1- and 3-day cultures. However, it has been observed that cells significantly proliferated on the PCLnp-Coll and PPCLnp-Coll bio-agents after 3 days compared to the control group. Importantly, after 3 days of culture, more than 80% of the cells were viable on both PCLnp-Coll and PPCLnp-Coll bio-agents, indicating very low cytotoxicity. It was also observed that cell growth on PCLnp-Coll and PPCLnp-Coll bio-agents increased significantly after 3 days of culture compared to PCL NPs (PCLnp) and PPCL NPs (PPCLnp), indicating an increase in biocompatibility following bio-functionalization with collagen. Finally, the results indicate that contact between cells and collagen on the surface of NPs affects cell viability, and PCLnp-Coll and PPCLnp-Coll bio-agents can induce cell proliferation.

## Conclusions

To reduce systemic exposure and increase PX retention time in the joint, following administration of IA, anti-inflammatory bio-agents consisting of PCL, collagen, and PX were constructed. The made bio-agent of PX-loaded PCL NP-Collagen (PPCLnp-Coll) showed a high swelling degree and low dissolution rate, which is why the drug was slowly and continuously released and only 63.97 ± 2.505% of the drug was released after 12 days. It seems that by the IA administration of the novel developed bio-agent, PX retention time will increase with sustained release of the drug and thereby increase the anti-inflammatory effect. On the other hand, the PPCLnp-Coll bio-agents were completely safe for the cells and their compatibility was confirmed by the analysis of the metabolic activity of the AT-MSCs through MTT assay. Therefore, the PPCLnp-Coll is expected to be applied in arthritic diseases, especially osteoarthritis due to the presence of biocompatible collagen and anti-inflammatory PX for drug delivery and osteochondral tissue engineering. Manufactured anti-inflammatory bio-agents can be used in the structure of implantable or injectable scaffolds for clinical uses.
